# Cloning, expression and enzyme activity delineation of two novel *CANT1* mutations: the disappearance of dimerization may indicate the change of protein conformation and even function

**DOI:** 10.1186/s13023-020-01492-8

**Published:** 2020-09-09

**Authors:** Hong-Dan Wang, Liang-Jie Guo, Zhan-Qi Feng, Da-Wei Zhang, Meng-Ting Zhang, Yue Gao, Chuan-Liang Chen, Bo-Feng Zhu

**Affiliations:** 1 Medical Genetic Institute of Henan Province, Henan Provincial People’s Hospital, Zhengzhou University People’s Hospital, Zhengzhou, 450003 People’s Republic of China; 2 National Health Commission Key Laboratory of Birth Defects Prevention, Henan Key Laboratory of Population Defects Prevention, Henan Institute of Reproduction Health Science and Technology, Zhengzhou, 450014 People’s Republic of China; 3 Clinical Research Center of Shaanxi Province for Dental and Maxillofacial Diseases, College of Stomatology, Xi’an Jiaotong University, Xi’an, 710004 People’s Republic of China; 4 College of Forensic Science, Xi’an Jiaotong University Health Science Center, Xi’an Jiaotong University, Xi’an, Shaanxi 710061 People’s Republic of China; 5 Department of Urology, The First People’s Hospital of Zhengzhou, Zhengzhou, 450004 People’s Republic of China; 6 Zhengzhou Orthopaedic Hospital, Zhengzhou, 450052 People’s Republic of China

**Keywords:** Desbuquois dysplasia, *CANT1*, Novel mutation

## Abstract

**Background:**

Desbuquois dysplasia (DBQD) was a rare autosomal recessive skeletal dysplasia. Calcium activated nucleotidase 1 (*CANT1*) mutation was identified as a common pathogenic change for DBQD type 1 and Kim variant but not for DBQD type 2. To our knowledge, all patients with DBQD type 1 currently found could be explained by mutations in the *CANT1* gene, but mutations in the *CANT1* gene might not be directly diagnosed as DBQD type 1.

**Results:**

We have identified two novel *CANT1* mutations (mut1: c.594G > A [p.Trp198*], mut2: c.734C > T [p.Pro245Leu]) in three children from a family of Chinese origin for the first time. Two of the three children could be diagnosed as typical DBQD type 1 and one child could not be diagnosed as DBQD type 1 based on the clinical data we had. To further clarify the effect of the two mutations of the *CANT1* gene, we studied the *CANT1* gene expression and detected the protein secretion and nucleotide enzyme activity through cDNA cloning and expression vectors construction for wild and mutant types. The mut1 was a nonsense mutation which could lead to premature termination and produced the truncated bodies; The *CANT1* dimer of mut2 was significantly reduced and even undetectable. The extracellular secretion of mut1 was extremely high while mut2 was significantly reduced compared with the wild type. And mut1 and mut2 also could result in a significant reduction in the activity of *CANT1* nucleotidease. From the results we could deduce that the two mutations of the *CANT1* gene were the causes of the two cases in this study.

**Conclusions:**

Regarding the particularity of the cases reported in this study, the pathogenesis of *CANT1* might be more complicated. The genetic and phenotype of three children with the same genetic background need to be further studied. Larger cohort of patients was needed to establish genotype–phenotype correlations in DBQD.

## Background

Desbuquois dysplasia (DBQD, OMIM#251450) was a rare autosomal recessive genetic disease with the incidence of approximately 1/1000000 and a lowest lethality rate of about 1/3 [1, 2]. In 1966, the clinical symptoms were first described by Desbuquois et al. [3]. The clinical symptoms of DBQD could be summarized as prenatal and postnatal growth retardation, short stature, multiple joint dislocations, scoliosis/lordosis, short long bones and hand abnormalities. Characteristic radiological features included advanced carpal bone age and a “Swedish key” appearance of the proximal femur. Additional anomalies of hand included distal phalangeal bifurcation or the delta phalanx of the first finger, accessory ossification center distal to the second metacarpal and phalangeal dislocations [4, 5].

In 2004, DBQD was classified into Type 1 (hand abnormalities present) and Type 2 (hand abnormalities absent) according to whether the above typical hand abnormalities occurs [6]. In 2010, Kim variant of DBQD has been distinguished characterized as having advanced carpal bone age, short metacarpals and elongated phalanges. The hand and spine abnormalities were the major clinical symptoms of this Kim variant [7]. The disease gene of DBQD was first located on chromosome 17q25.3 by a genome-wide research in four DBQD families with DBQD type 1 patients in 2003 [8]. Until 2009, calcium-activated nucleotidase 1 (*CANT1*) mutations were first identified in patients of DBQD type 1 [9]. In the studies after 2009, *CANT1* mutations were supported as the causes of DBQD type 1. The studies also found that the *CANT1* mutations were responsible for Kim variant and atypical DBQD type 2 [4, 10]. For DBQD type 2, xylosyltransferase 1 (*XYLT1*) mutations were found in some of the patients. There were still some unknown molecular bases of DBQD type 2 [11, 12]. To summarize, *CANT1* mutation was a common pathogenic change for DBQD type 1 and Kim variant but not for DBQD type 2.

At present, we identified two novel *CANT1* mutations (compound heterozygous) in three children from a family of Chinese origin. According to their clinical symptoms, two of the three children could be diagnosed as typical DBQD type 1. The other child only had height restrictions with a height of 142 cm in her 16 years who couldn’t be diagnosed as DBQD based on the clinical data. This study found the genetic heterogeneity of *CANT1* which has not been reported in previous studies. Through the in vitro experiments by synthesizing full-length wild-type and mutant cDNA of *CANT1* gene, this research still supported the previous findings that *CANT1* mutations were responsible for DBQD type 1. The results of our in vitro experiments showed that the disappearance of dimerization of the mutant protein might be a change affecting *CANT1* enzyme activity.

## Results

### Clinical report

**Fig. 1:**
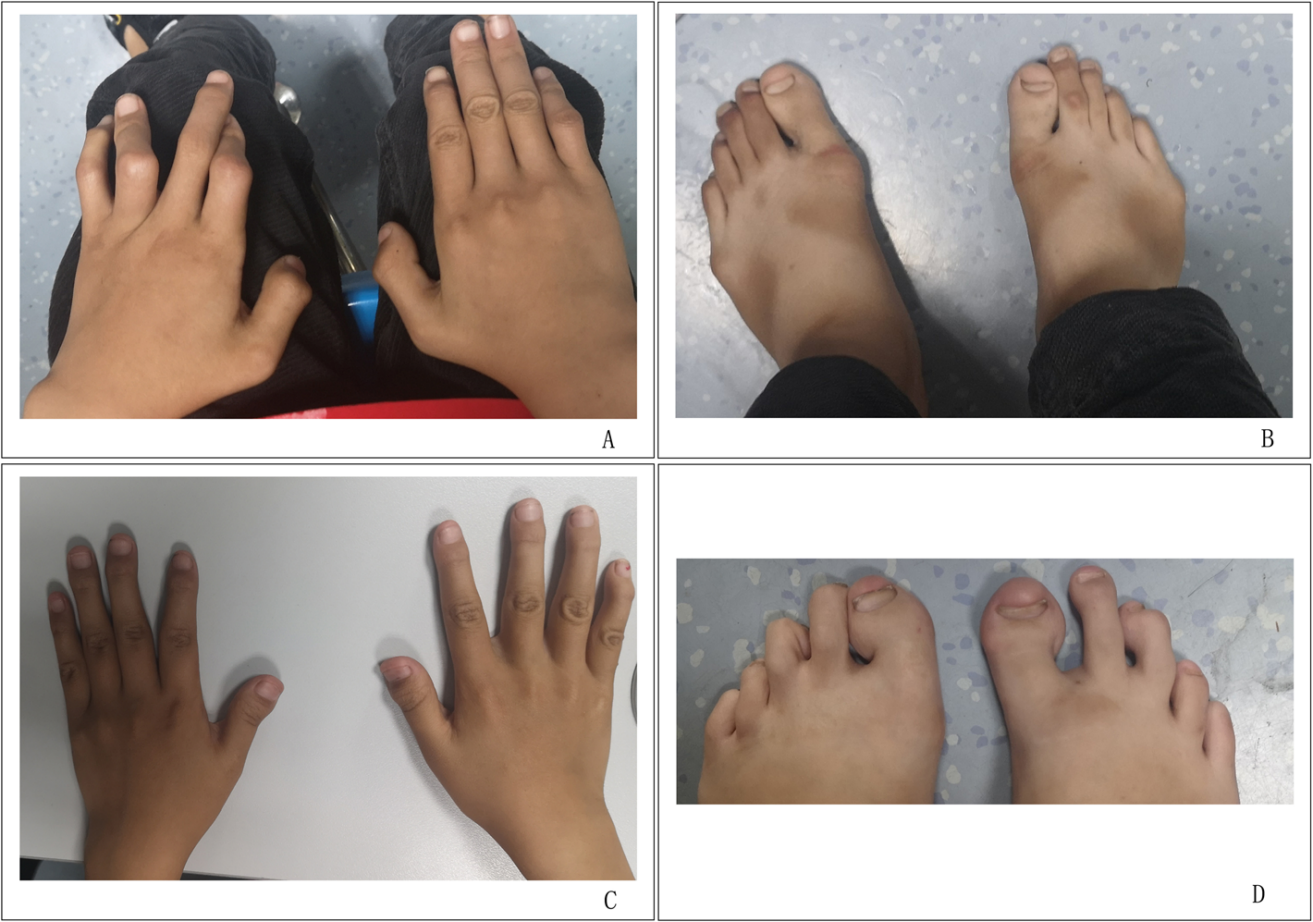
The clinical photographs of the hands and feet of child 2 and 3. **a** and **b**: Child 2 at age 13 years, male; there were abnormal curvature of the fingers (toes) in both hands (feet); **c** and **d**: Child 3 at age 8 years, female; there were abnormal curvature of the fingers (toes) in both hands (feet)

**Fig. 2:**
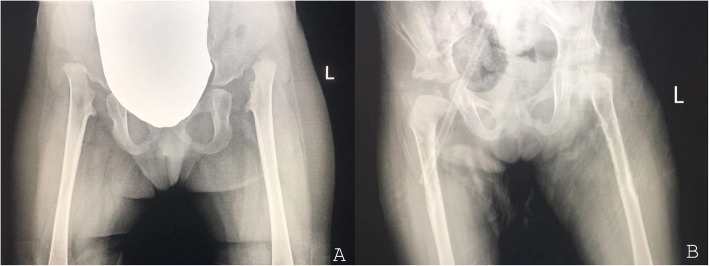
Radiographs of the hip of child 3. **a**: Hip joint at 3 years old, Swedish key appearance could be observed; **b**: Hip joint after operation at 4 years old, the postoperative prognosis is good

**Fig. 3:**
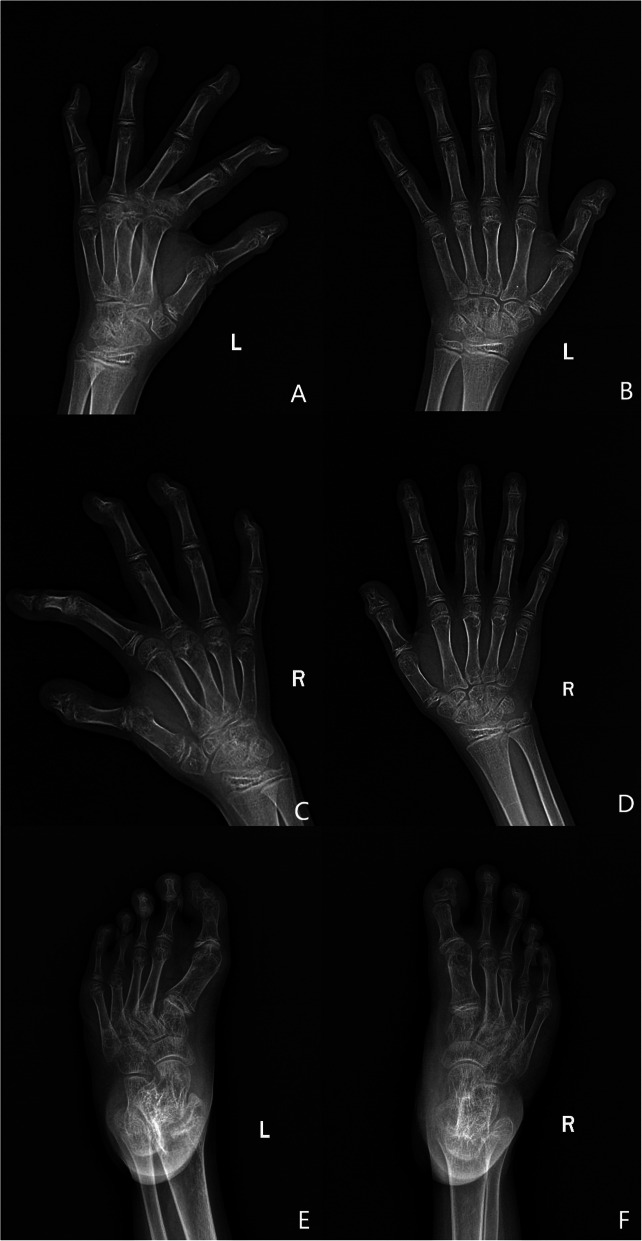
Radiographs of the hands and feet of child 3 aged 8 years. **a** and **b**: Left hand, dislocation and advanced carpal bone ages could be observed; **c** and **d**: Right hand, dislocation, bifurcation of the distal phalanx of the first finger and advanced carpal bone ages could be observed; **e** and **f**: Feet, dislocation could be observed

The parents of the patients came to the clinic of the medical genetics institute of Henan Provincial People’s Hospital for genetic consultation in order to have guidance to aristogenesis and good brood. The parents were pregnant three times and gave birth three times in full term. The clinical phenotype of parents was normal. The clinical manifestations and radiological findings of the 3 children were showed in Table 1. The first child (child 1) was only limited in height (142 cm, Female, 16 years) and had normal intelligence. And she had no clinical symptoms such as joint dislocation and heart function limitation since birth. The second child (child 2) had pre and postnatal growth retardation, dislocation of joint, lumbar vertebra scoliosis, Mitral valve prolapse with severe insufficiency, camptodactyly, claw toes and hammer toes (Fig. 1a and b). At the age of 3 years, the operation was performed because of congenital dislocation of both hip joints and postoperative recovery was well. At the age of 7 years and 10 months, “mitral valvuloplasty” was performed for mitral valve prolapse with insufficiency found in physical examination and postoperative recovery was well. The third child (child 3) had pre and postnatal growth retardation, dislocation of joint, mitral insufficiency, atrial septal defect, camptodactyly, claw toes and hammer toes (Fig. 1c and d). From her 16 months to 2 years old, the operations were performed due to dislocation of hip joint and postoperative recovery was well. At the age of 2 years and 11 months, mitral valvuloplasty and atrioseptopexy was performed for mitral insufficiency and atrial septal defect. We retrieved radiographs of the hip dislocation of the third child before surgery. Hip dislocation and typical Swedish key appearance could be observed (Fig. 2). We reviewed the X-ray of her hands and feet at her 8 years old (Fig. 3). The radiographs showed bifurcation in the distal phalanx of the first finger of right hand, Metacarpophalangeal (mp) joint dislocation, distal finger joint dislocation, the bone line closure in the distal phalanx of the two hands and the advanced carpal bone ages which were the typical hand manifestations of the DBQD type 1. From clinical symptoms and radiological findings we can diagnose these two patients as DBQD type 1.

**Table 1:** Clinical Manifestations and radiological findings of the 3 children

Patient ID	Origin	weeks of gestation	Birth Weight	Sex	Age	Height/Weight	Dislocation of joint	Hand	Heart	Spine	Orthopedic surgery	Heart surgery	Mutations
Child 1	Chinese Han	Term delivery	?	Female	16 years	142 cm/32 kg	–	?	?	?	–	–	c.594G > A and c.734C > T
Child 2	Chinese Han	Term delivery	2.4 kg	Male	13 years	?/24 kg	Hip, Metacarpophalangeal (mp), distal finger	Bifurcation of the distal phalanx of the first finger	Mitral valve prolapse with severe insufficiency	Lumbar vertebra scoliosis	+	+	c.594G > A and c.734C > T
Child 3	Chinese Han	Term delivery	2.2 kg	Female	8 years	?/18 kg	Hip, Metacarpophalangeal (mp), distal finger (toe)	Bifurcation of the distal phalanx of the first finger	Mitral insufficiency, Atrial septal defect	–	+	+	c.594G > A and c.734C > T

*?: unknow; −: no related performance; +: have related performance

### Identification of the novel compound heterozygous mutations of the *CANT1* gene

We examined the 3 children and their parents by using WES. Based on the autosomal recessive inheritance, types of the mutations and minor allele frequency, the variants were filtered. Novel compound heterozygous mutations of the *CANT1* gene were found in the 3 children (c.594G > A [p.Trp198*], c.734C > T [p.Pro245Leu]). Two loci were heterozygous mutations located in the second and third exons, respectively.

**Fig. 4:**
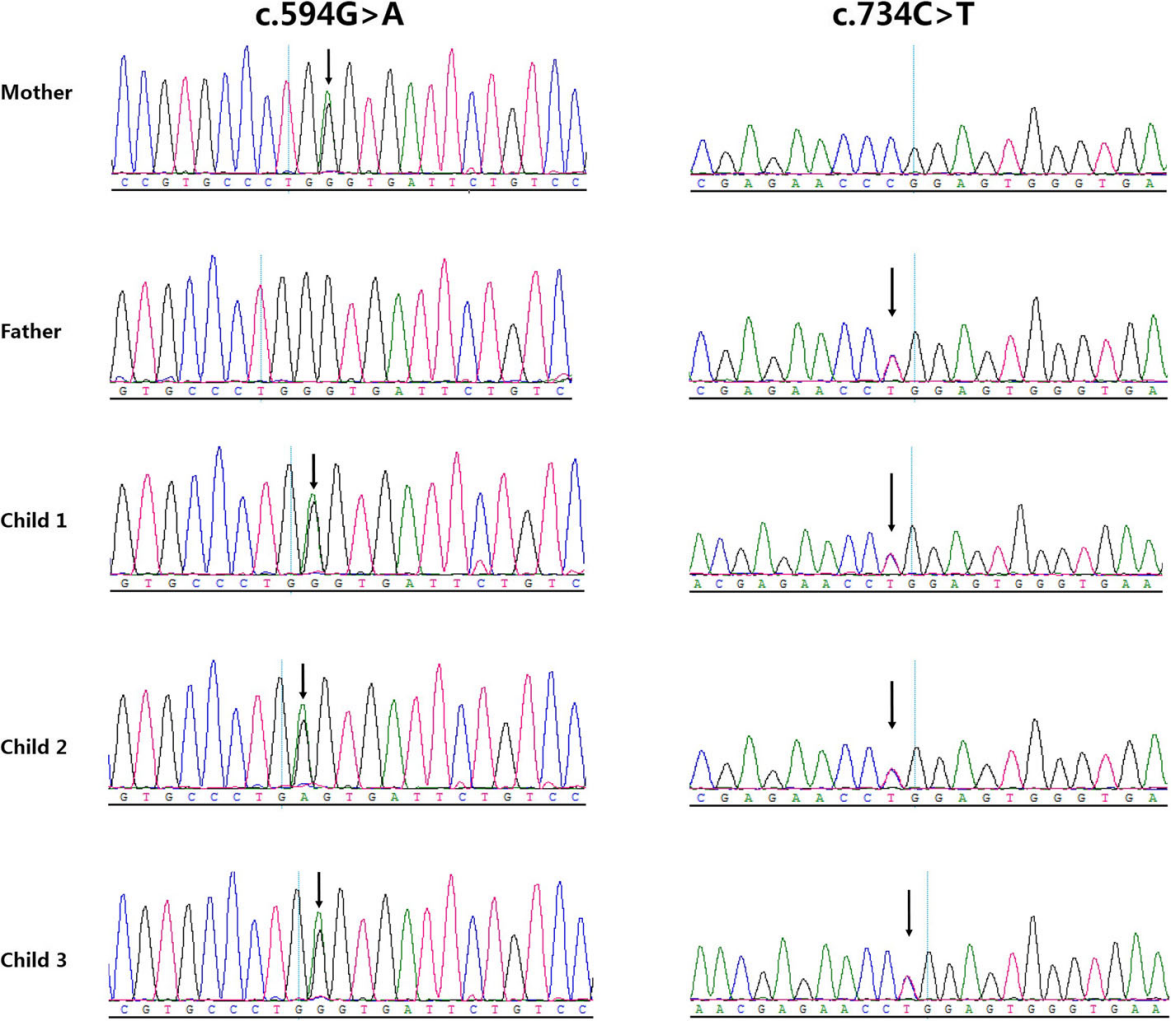
Sanger sequencing map of the mutations in the *CANT1* gene from the parents and their three children

The mutations of the *CANT1* gene were then confirmed by direct sequencing. From the sequencing results, we could see that the mutation c.594G > A of the 3 children was inherited from the mother. Mutation c.734C > T was inherited from the father. It could be concluded that the mutations were compound heterozygous mutations and the genetic phenotypes were co separated (Fig. 4).

### The effect of mutations on the *CANT1* gene expression

**Fig. 5:**
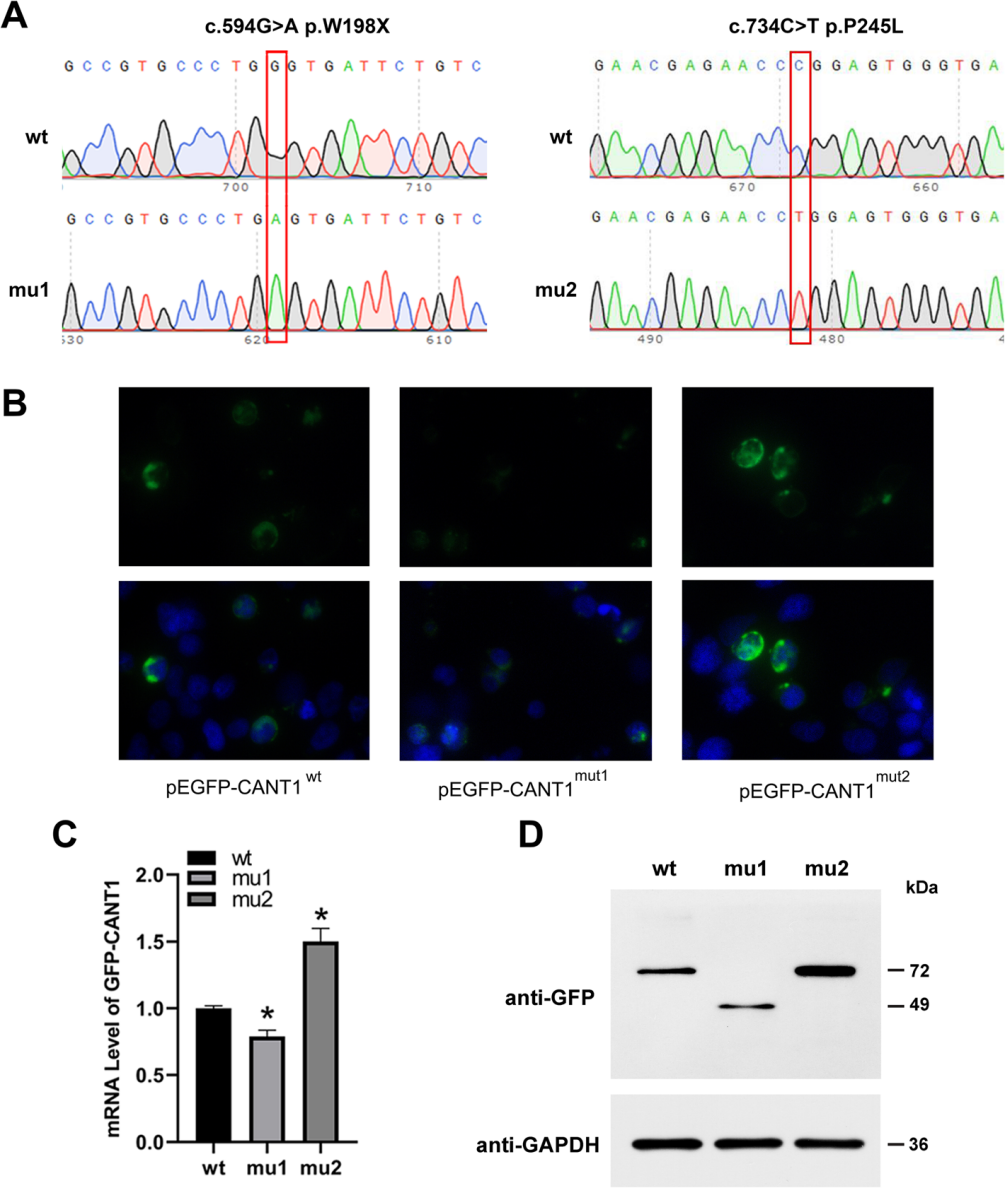
Mutations c.594G > A (p.W198X) and c.734C > T (p.P245L) caused changes in the expression level of the *CANT1* gene. **a**: Sequencing results showed that mutations c.594G > A (p.W198X) and c.734C > T (p.P245L) were successfully introduced; **b**: IF results (measurement scale was 40 times); **c**: mRNA expression detected by qPCR; **d**: *CANT1* protein expression results by WB

The constructed eukaryotic expression vector pEGFP-C1 *CANT1* wt / mut1 (c.594G > A)/mut2 (c.734C > T) was transfected into HEK293T cells for 48 h and then detected (Fig. 5a). Photographs were taken with a GFP fluorescence microscope, and it was found that the wild-type *CANT1* protein was localized mainly in the nucleus. The GFP signal of mut1 was significantly weakened, while the GFP signal of mut2 was significantly enhanced and obvious localization was visible in the nucleus (Fig. 5b).

qPCR was used to detect the mRNA expression of wild-type and mutant genes in HEK293T cells. The results showed that the expression of the *CANT1* mut1 was significantly reduced and the expression of the *CANT1* mut2 was significantly increased. It showed that the mutations affected the expression of *CANT1* gene at the transcription level (Fig. 5c).

The Western blotting (WB) results were showed in Fig. 5d. Compared to the wild-type *CANT1* (72 kDa, GFP-*CANT1*-wt), mut1 was a nonsense mutation leading to premature termination (49 kDa, GFP-*CANT1*-mut1), producing truncated bodies. The protein expression of mut1 was significantly reduced; while mut2 did not lead to premature termination (72 kDa, GFP-*CANT1*-mut1) and the protein expression was significantly increased.

### Detection of *CANT1* dimerization

**Fig. 6:**
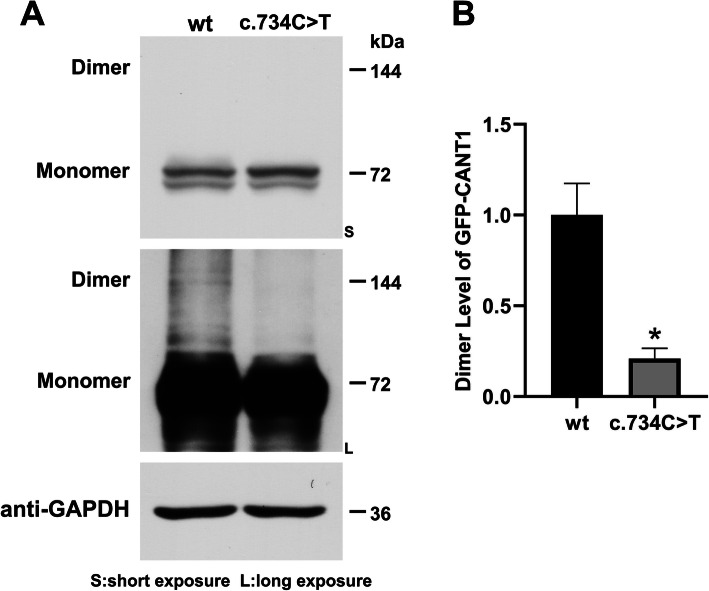
The mutation c.734C > T (p.P245L) caused the *CANT1* dimer to depolymerize. **a**: the expression of *CANT1* dimerized protein was detected by WB. **b**: the WB gray scan results of *CANT1* dimerized protein expression

The non-reducing WB method was used to detect the difference of *CANT1* wt / mut2 dimerization. The constructed eukaryotic expression vector pEGFP-C1 *CANT1* wt/mut2 was transfected into HEK293T cells. After 36 h of transfection, 2 mM Ca^+^ was added to stimulate and samples were collected for WB detection. The results showed that the wild-type *CANT1* had a band at 144 kDa, indicating that dimerization could occur. The mutant *CANT1* dimer was significantly reduced and even undetectable by WB (Fig. 6a). Quantitative results after scanning of gray values suggested that *CANT1* mut2 could affect the occurrence of *CANT1* dimers (Fig. 6b).

### Detection of protein secretion and nucleotide enzyme activity

**Fig. 7:**
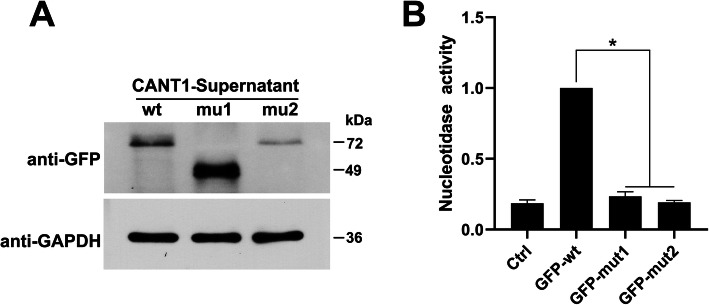
The two mutations affect the extracellular secretion and nucleotide activity of *CANT1*. A: the expression of the extracellular secreted *CANT1* protein was detected by WB; B: the result of *CANT1* nucleotide enzyme activity

We collected the supernatants of the cells and detected the difference in the content and activity of *CANT1* wt/mut1/mut2 protein secreted extracellularly by WB and molybdenum blue colorimetry. WB results showed that compared with the secreted protein of wild-type *CANT1* (72 kDa, GFP-*CANT1*-wt), the nonsense mutation mut1 (c.594G > A) also occurred extracellular secretion, and its secretion was extremely high. The secretion of mut2 (c.734C > T) was significantly reduced (Fig. 7a). The results of nucleotide enzyme activity showed that mutations of mut1 (c.594G > A) and mut2 (c.734C > T) resulted in a significant reduction in the activity of *CANT1* nucleotidease (Fig. 7b).

### 2D and 3D structure of *CANT1* (Mut 2: p.Pro245Leu)

**Fig. 8:**
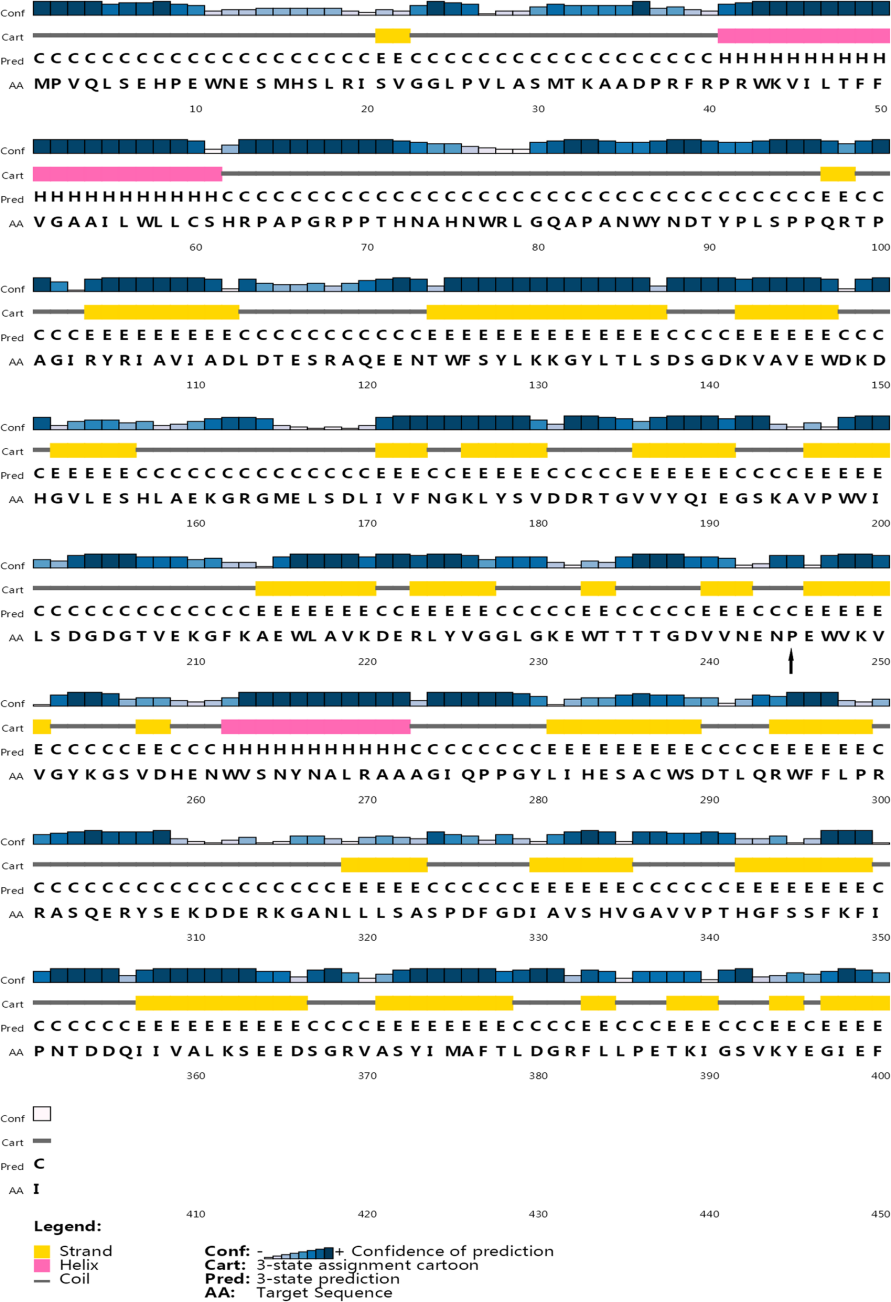
2D structure of *CANT1* (p.Pro245Leu), and arrow indicated mutation position

**Fig. 9:**
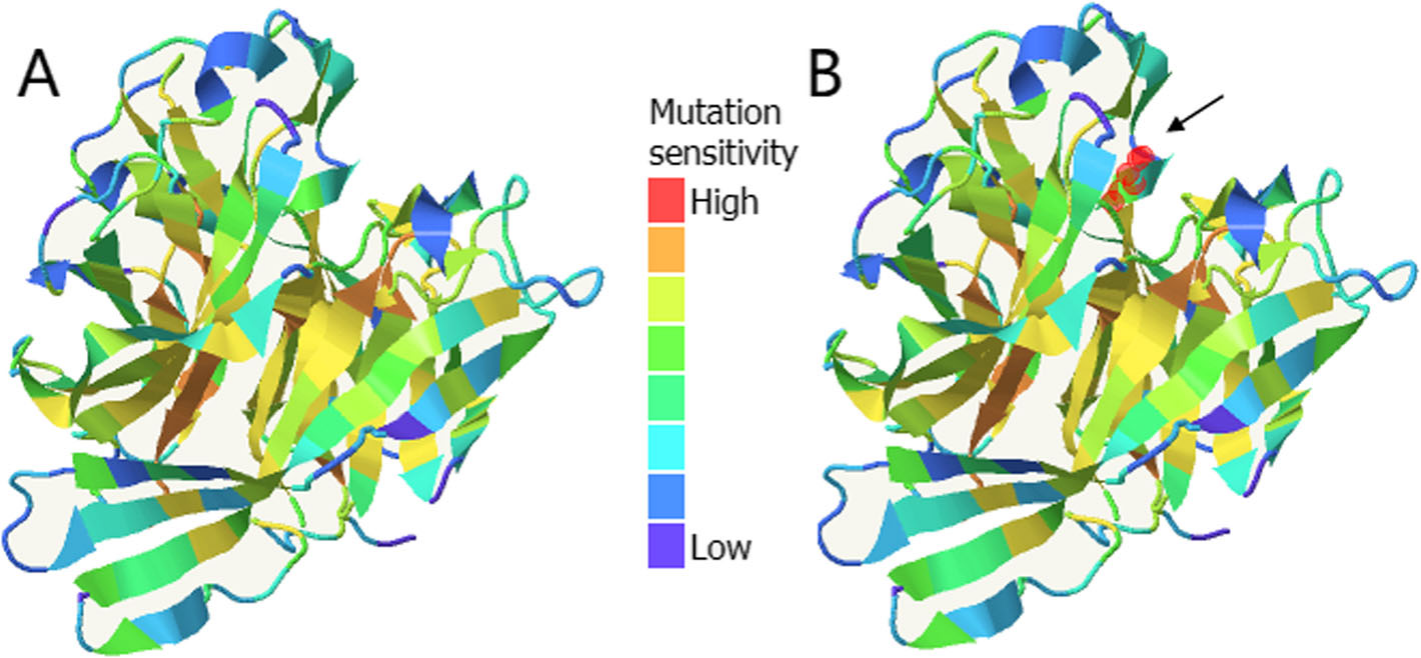
3D structure of *CANT1* (p.Pro245Leu). **a**: the mutation sensitivity diagram of CANT1 protein; **b**: the mutation sensitivity diagram of *CANT1* protein showing Pro245 position in orange and marked with arrow

2D structure of *CANT1* (p.Pro245Leu) was showed in Fig. 8. We could see that the Pro245 residue was involved in the coil of the 2D structure and the prediction reliability was high. It was a highly conserved residue located on a conserved 2D fragment [13]. Mutations in this residue might affect the function of the human *CANT1* protein. 3D structure of *CANT1* (p.Pro245Leu) was showed in Fig. 9. On the left was the mutation sensitivity diagram of *CANT1* protein (Fig. 9a). On the right was the mutation sensitivity diagram of *CANT1* protein showing Pro245 position and the mutation position was shown in orange and marked with arrow (Fig. 9b). Positions were colored according to the average effect of the 20 possible mutations at that position. According to the color, we could predict whether the missense mutations in the protein were likely to have a functional/phenotypic effect. It was divided into 8 levels according to the color column in the middle of the figure, and the mutation sensitivity of Pro245 was at the 5th level. Proline was a neutral amino acid which was a cyclic sub-amino acid and its solubility in water was greater than other amino acids. Leucine was a branched-chain amino acid which was almost insoluble in water. Changes in amino acid structure and hydrophilicity might affect the protein activity.

## Discussion

DBQD was classified as the multiple dislocation group according to nosology and classification of genetic skeletal disorders [1]. Clinical symptoms included multiple joint dislocations, joint laxity, scoliosis/lordosis, severe pre and postnatal growth retardation. As has been noted, DBQD type 1 and type 2 were distinguished according to the presence or absence of unique hand abnormalities [6]. The Kim variants were characterized by accelerated carpal bone ages, short metacarpals and elongated phalanges (nearly equal length of the second to fifth fingers). Severe precocious ostarthritis of the hands and spine was a major characteristic finding of the Kim variants [7]. As far as I known, 36 cases of DBQD have been reported and the *CANT1* mutations were found in almost all the DBQD type 1 patients [4, 9, 10, 14–18]. There were 27 distinct *CANT1* mutations were found, including 12 missense mutations, 11 exon region frame shift mutations, 2 nonsense mutations, 1 intron frame shift mutation and 1 splice site mutation. All three types of DBQD could be observed only in the Turkish population. Until now, the Kim variant has only appeared in Japan, Korea and India which belonged to Asia and Turkish population. *CANT1* mutations were supported as the causes of DBQD type 1 patients, Kim variant and atypical DBQD type 2 (only two cases). There were still some unknown molecular bases of DBQD type 2. It was important to correctly diagnose and type the DBQD based on the patient’s clinical symptoms rather than relying solely on genetic mutation information.

Here we reported two novel *CANT1* mutations (compound heterozygous) in three children from a family of Chinese origin. Two of them (child 2 and 3) could be diagnosed as DBQD type 1 and the remaining child (child 1) could not currently be diagnosed with clinical symptoms. The parents have proven to be the biological parents of the children by short tandem repeats (STR). The STR results were showed in Supplementary Fig. 1. We found cardiac mitral valve prolapse in both patients, which has not been found in previous reports of DBQD type 1 patients. Unfortunately, we did not get more clinical symptoms and physical examination results for the child 1. According to the parents’ statement, no symptoms such as joint dislocation and cardiac abnormalities occurred, and there was no history of surgery of child 1. According to previous related reports, we believed that the mutations of *CANT1* might to be the cause of the disease in these two patients. In this case, it was particularly important to study the function of the two mutations of *CANT1*.

Until now, the molecular mechanism of DBQD caused by *CANT1* gene was less studied. The *CANT1* gene could form 12 transcript variants. The gene of transcript variant 1 was formed by exon 1, 2, 3 and 4 which had 8 nucleotidase conserved regions (NCR) [13]. In this study, novel compound heterozygous mutations of the *CANT1* gene were found in the family (mut1: c.594G > A [p.Trp198*], mut2: c.734C > T [p.Pro245Leu]) and located in the second and third exons, respectively. Mut1 was located in NCR3 (Exon 2) which was highly conserved and was the same in all the selected 11 sequences (Supplementary Fig. 2 A and B). Mut1 caused the formation of a stop codon, and the protein was truncated at position 198 which will likely affect NCR3–8, destroy important conserved structures of the protein, and might have an impact on the protein function. Mut2 was located in NCR5 (Exon 3) which had the same residue in all the selected 11 sequences and was as conservative as mut1 (Supplementary Fig. 2 A and B). In addition, a homozygous mutation of c.734 delC [p.P245RfsX3] has been found in a DBQD type 1 patient from Turkey and the mutation was lethal [9]. Mut2 was also occurred at this position which might mean that this locus was important for the function of the protein.

*CANT1* was a soluble nucleotide enzyme belonged to apyrase family which could preferentially hydrolyze UDP next GDP and UTP [19, 20]. However, the exact function of the protein was still unknown [21]. In order to further understand the function of human *CANT1* protein, we constructed eukaryotic expression vector pEGFP-C1 *CANT1* wt/mut1 (c.594G > A)/mut2 (c.734C > T) and transfected into HEK293T cells. We studied the effect of mutations on the *CANT1* gene expression, protein secretion and nucleotide enzyme activity. For mut1, it could be seen from the results that truncated protein was formed, the amount of distribution around the nucleus and activity were decreased and extracellular secretion was increased. Previous research showed that the activity of this family was strictly dependent on calcium, and the calcium could cause conformational changes of the secreted, soluble human nucleotidase protein [22]. The truncated protein formed by mut1 might lose the critical calcium-binding region for activity which was also critical region responsible for forming dimerization through covalent binding, but retain the region responsible for extracellular secreted. For mut2, there were some phenomena different from mut1. It could be seen that the expression was increased, dimerization was disappeared, the amount of distribution around the nucleus was increased, and extracellular secretion and activity were decreased. The increase in expression might be due to the disappearance of dimerization, rather than a true increase in expression. Pro245 might be a critical region for calcium binding. And the mutation affected the conformation of the protein, leading to changes in its secretion and activity. In addition, the prediction of the 2D and 3D structure of *CANT1* showed that Pro245 was in an important 2D structure (coil) and was located in a superficial position of the protein. The highly hydrophilic cyclic amino acid proline was replaced by the low hydrophilic branched chain amino acid leucine which will definitely affect the function of the protein.

Previous studies have shown that mutations in the *CANT1* gene may be related to impaired endoplasmic reticulum function [9], and may also affect Golgi function and affect proteoglycan synthesis [4]. Other study has shown that *CANT1* was found to be a target gene of the DREAM protein involved in the process of protein folding and degradation [23]. In short, *CANT1* gene mutations might cause a defect in the *CANT1* protein by affecting *CANT1* protein secretion, conformation, etc. and affect the protein biosynthesis process. Recently, *CANT1* knock-in and knock-out mice have been successfully prepared and the DBQD type 1 phenotype was recapitulated [24]. This provided individual phenotypic evidence of the important role of the gene in cartilage ossification and the synthesis of cartilage proteoglycan.

## Conclusions

In this study, we reported two novel *CANT1* mutations in three children from one family. It was worth pointing out that two of the three could be diagnosed as typical DBQD type 1 according to their clinical symptoms. At the same time, a new symptom (mitral valve prolapse) different from previous reports was observed. However, the other child only had height restrictions with a height of 142 cm in her 16 years who couldn’t be diagnosed as DBQD based on our clinical data. Based on the above, we could consider that the mutations of the *CANT1* gene were the cause of the two cases of DBQD type 1 in this study. We haven’t been able to give a reasonable explanation as to why the child 1 did not have symptoms of DBQD type 1. The substrates (UDP, GDP and UTP) of the *CANT1* protein were involved in the functional regulation of several important signaling molecules, which showed not only the importance of *CANT1* but also the complexity of its role. This might explain the genetic heterogeneity present in this study. The genetic and phenotype of three children with the same genetic background need to be further studied, and the pathogenesis of *CANT1* may be more complicated.

## Materials and methods

### Patients and clinical assessment

The patients were identified and collected through clinical genetics counseling services at the clinic of Henan Provincial People’s Hospital. The parents on behalf of the children signed the informed consent for the study. The study was approved by the human and ethics committee of Henan Provincial People’s Hospital.

There are 5 members in this family, including parents with normal phenotype and 3 children with abnormal phenotype. The healthy parents of Chinese Han descent were not close relatives and had no history of adverse contact during pregnancy. The clinical manifestations and radiological findings of the 3 children were summarized in Table 1. According to the diagnostic criteria for DBQD type 1 and the clinical manifestations of the children, children 2 and 3 could be diagnosed as DBQD type 1. The clinical manifestations for these two patients consistent with DBQD type 1 included: pre and postnatal growth retardation, dislocation of joint, Swedish key appearance of the proximal femur and characteristic hand anomalies (Figs. 1, 2 and 3).

### DNA extraction, whole exome sequencing and data analysis

The peripheral blood samples were obtained from the couple and their three children. Genomic DNA was extracted from 500 μl peripheral bloods using a standard commercial kit (TIANGEN, Beijing, China) following the manufacturer’s instructions. The quantity of the DNA samples was determined using Qubit dsDNA HS assay kit on Qubit 3.0 (Invitrogen Co., California, USA).

Whole Exome Sequencing (WES) was conducted using the AIExomeV2 kit (iGeneTech Co., Beijing, China). According to the manufacturer’s standard operation protocol, the whole exon region was enriched by liquid phase probe method and sequenced on Illumina Nova sequence platform (Illumina, Inc., California, USA). 1% was used as Minor allele frequency (MAF) threshold in this study. The depth used in the WES was 100× and the coverage of targeted exons was 99%.

### Mutation confirmation

In order to determine the mutations and identify whether the mutations co-segregated with the disease phenotype the 5 family members were sequenced and analyzed using ABI3500 Genetic Analyzer and Sequencing Analysis software (Applied Biosystems, Foster City, CA, USA). Specific sequences containing the two mutations were amplified and Primer 5 software was used for designing the primers. The primers were showed in Supplementary Table 1.

### cDNA cloning and construction of expression vectors

Target cDNA synthesis was completed by Wuhan Shishi Biopharm Co. Ltd. using gene splicing by overlap extension PCR method (SOE PCR). Using human *CANT1* cDNA as a template, an expression vector was constructed: the entire length of the *CANT1* cDNA was cloned into the pEGFP-C1 plasmid through restriction sites Xho I and BamH I to construct a recombinant vector pEGFP-C1 *CANT1* wt (wild type) (Wuhan Shishi Biopharm Co., Wuhan, China). Using site-directed mutagenesis, the mutations c.594G > A [p.Trp198*] and c.734C > T [p.Pro245Leu] were introduced into the above-mentioned recombination vectors, respectively, and the recombinant vectors pEGFP-C1 *CANT1* mut 1 and pEGFP-C1 *CANT1* mut 2 were constructed. This set of vectors expressed the fusion protein GFP *CANT1*.

### Cell transfection and immunofluorescence (IF) staining

HEK293T cells were cultured in DMEM medium (Gibco, Thermo Fisher) containing 10% fetal bovine serum, and the constructed wild-type and mutant eukaryotic recombinant expression vectors were transiently transfected into HEK293T cells according to the liposome instructions of Lipo2000 (YEASEN, Shanghai, China). Forty-eight hours after transfection, wild-type and mutant fluorescent pictures were taken and qPCR tests were performed after collection. SYBR Green Realtime PCR Master Mix was used for qPCR (TOYOBO Co., Shanghai, China).

### RNA analysis

Cell samples were collected 48 h after transfection of wild-type and mutant recombinant expression vectors. Total RNA was extracted by Trizol method (Invitrogen, Gaithersburg, MD, USA). cDNA synthesis was performed using a commercialization Kit according to the instructions (Takara, Dalian, China). And the expression difference of wild-type and mutant target genes was detected by qPCR by ABI StepOne Real-Time PCR system (ABI, Vernon, USA). SYBR Green Realtime PCR Master Mix was used for qPCR (TOYOBO Co., Shanghai, China).

### Western blotting (WB)

After 36 h of the wild-type and mutant recombinant expression vectors transfection, the medium was replaced with serum-free medium Opti-MEM and the culture was continued for 36 h to collect the supernatant and cell pellet. Use Amicon® Ultra-4 Centrifugal Filters to process the cell supernatant at 4 °C and 3000 rpm, and concentrate 2 mL of the supernatant to 200ul to extract the secreted proteins. RIPA lysate was used to extract total protein from the cell pellet. The BSA kit was used to determine the protein concentration, followed by protein denaturation treatment, and the WB method was used to detect the difference in protein expression of wild-type and mutants and the degree of dimerization of the target proteins.

Non-reduced WB was used in this study. The method of non-reduced WB was basically the same as that of conventional WB, but fresh and sufficient protease inhibitor (Pierce) was added to the cell lysate, and the reducing agent components such as SDS must be removed from the cell lysate, loading buffer, and PAGE gel.

### Nucleotidase activity assay

The treatment of cell culture supernatant was as follows. Concentrate 1 mL of the supernatant to 100ul to extract the secreted proteins. The 100ul supernatant was diluted 10 times with 40 mM succinate buffer (pH 6.5, containing 4 mM CaCl _2_, 2 mM UDP), and incubated at 37 °C for 1 min. The inorganic phosphorus detection kit was used to detect the free phosphorus Pi content using ferrous sulfate molybdenum blue microplate method according to the instructions, and the *CANT1* nucleotide enzyme activity was calculated.

### Prediction of 2D and 3D structure of *CANT1* (Mut 2:p.Pro245Leu)

The homologous sequence of human *CANT1* protein was extracted from NCBI-Homologene Database and used an online website PredictProtein (http: //www.predactprotein) predicted the 2D structure of *CANT1* [25]. The online website Phyre (www.sbg.bio.ic.ac.uk/phyre) was used for predicting the 3D structure of *CANT1* [26].

## Supplementary information

**Additional file 1: Supplementary Figure 1.** The STR results of the 5 family members. A: Mother; B: Father; C: Child 1; D: Child 2; E: Child 3.

**Additional file 2: Supplementary Figure 2.** The gene structure and the genomic alignment of the *CANT1* gene. A: The diagram of Exon-Intron-Mutation of the *CANT1* gene; B: The genomic alignment for mut 1 and mut 2 conservation of 11 *CANT1* Homologous sequences.

**Additional file 3: Supplementary Table 1.** The sequence of the primers used in mutation confirmation.

## Data Availability

All data could acquire from the corresponding author if reasonably required.

## Abbreviations

DBQD: Desbuquois dysplasia
*CANT1*: Calcium activated nucleotidase 1
IF: Immunofluorescence
WB: Western blotting
