# Supplementary figures and images for: Cloning, expression and enzyme activity delineation of two novel CANT1 mutations: the disappearance of dimerization may indicate the change of protein conformation and even function
Source: Orphanet J Rare Dis. 2020 Sep 9;15:240. doi: 10.1186/s13023-020-01492-8 (PMC7487677; doi:10.1186/s13023-020-01492-8)

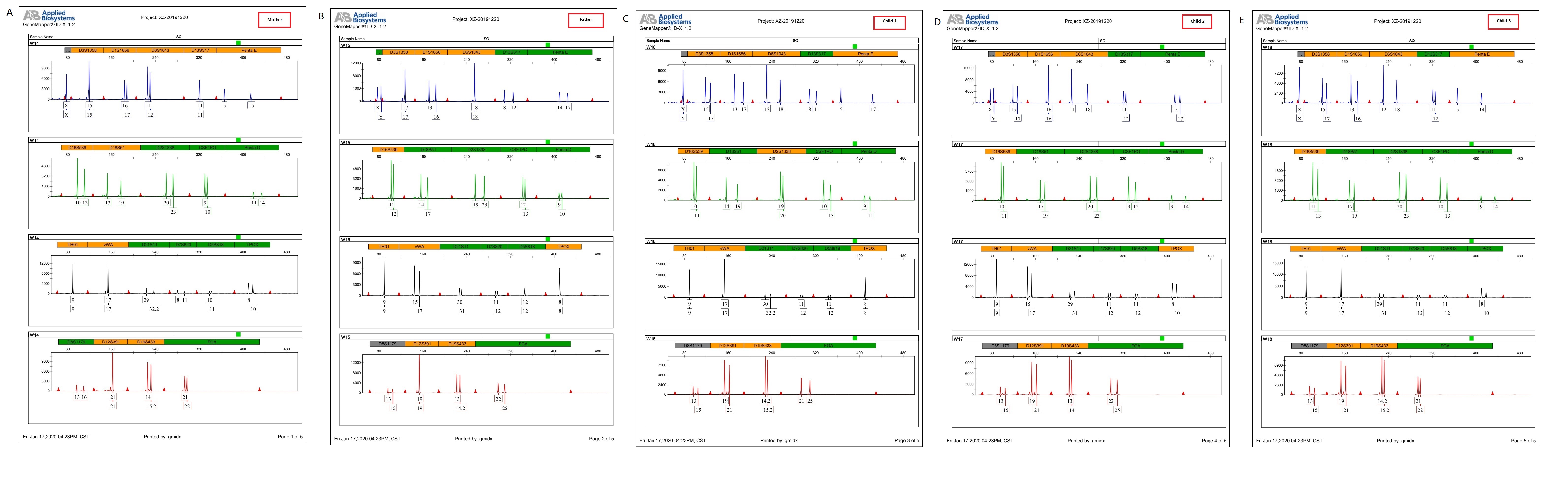

Supplement: Supplementary file 1 — Additional file 1: Supplementary Figure 1. The STR results of the 5 family members. A: Mother; B: Father; C: Child 1; D: Child 2; E: Child 3. [file 13023_2020_1492_MOESM1_ESM.jpg]

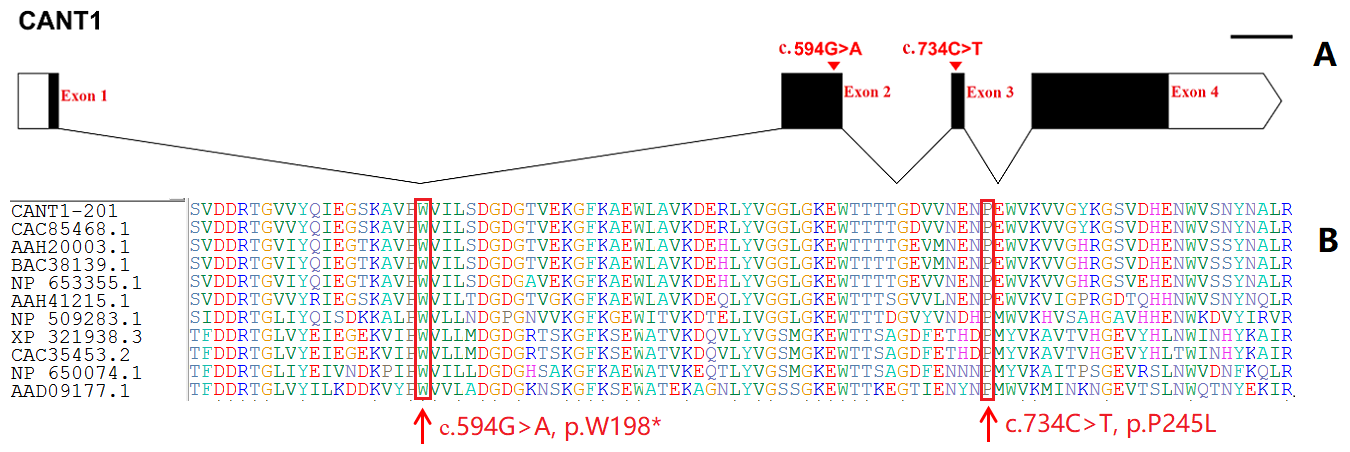

Supplement: Supplementary file 2 — Additional file 2: Supplementary Figure 2. The gene structure and the genomic alignment of the CANT1 gene. A: The diagram of Exon-Intron-Mutation of the CANT1 gene; B: The genomic alignment for mut 1 and mut 2 conservation of 11 CANT1 Homologous sequences. [file 13023_2020_1492_MOESM2_ESM.png]
