# Supplementary material for: Cloning, expression and enzyme activity delineation of two novel CANT1 mutations: the disappearance of dimerization may indicate the change of protein conformation and even function
Source: Orphanet J Rare Dis. 2020 Sep 9;15:240. doi: 10.1186/s13023-020-01492-8 (PMC7487677; doi:10.1186/s13023-020-01492-8)
Supplement: Supplementary file 3 — Additional file 3: Supplementary Table 1. The sequence of the primers used in mutation confirmation. [file 13023_2020_1492_MOESM3_ESM.docx]

Supplementary Table 1. The sequence of the primers used in mutation confirmation.

| Primer name | Primer sequence (5'-3') |
| --- | --- |
| c.594G> A (F) | AAACTCTACTCCGTGGATGACCGG |
| c.594G> A (R) | GATACAGCCGAAAACATCTTGGAG |
| c.734C> T (F) | TAATCCTAAGGTTGGGGTTACAGC |
| c.734C> T (R) | CGTTGTAGTTGGACACCCAGTTCT |
